# Supplementary material for: NULISA: a proteomic liquid biopsy platform with attomolar sensitivity and high multiplexing
Source: Nat Commun. 2023 Nov 9;14:7238. doi: 10.1038/s41467-023-42834-x (PMC10636041; doi:10.1038/s41467-023-42834-x)
Supplement: Supplementary file 1 — Supplementary Information [file 41467_2023_42834_MOESM1_ESM.pdf]

Supplementary Information for

“NULISA: a proteomic liquid biopsy platform with attomolar sensitivity and high multiplexing”

by Feng et al.

Supplementary Tables

Supplementary Table 1. Sample sets and disease group categories used in NULISAseq verification studies

Disease type	Primary diagnosis	Sample Set 1 n=74	Sample Set 2 n=77
Healthy (n=79)		39	40
Total disease (n=72)		35	37
Cancer (n=32)	Bladder cancer (n=2)	1	1
	Breast cancer (n=2)	2	0
	Cervical cancer (n=1)	0	1
	Colorectal cancer (n=1)	1	0
	Gastric cancer (n=1)	0	1
	Head & neck cancer (n=5)	3	2
	Leukemia (n=3)	2	1
	Lung cancer (n=5)	2	3
	Lymphoma (n=6)	3	3
	Melanoma (n=1)	0	1
	Myeloma (n=3)	1	2
	Pancreatic cancer (n=2)	2	0
Inflammatory disease (n=21)	Rheumatoid arthritis (n=5)	2	3
	Sjögren's Syndrome (n=5)	3	2
	Systemic lupus erythematosus (n=5)	3	2
	Ulcerative colitis (n=6)	4	2
Kidney disease (n=4)	Chronic kidney disease (n=4)	2	2
Metabolic disease (n=9)	Type I diabetes (n=7)	3	4
	Type II diabetes (n=2)	1	1
Neurological disease (n=6)	Alzheimer’s disease (n=1)	0	1
	Parkinson's disease (n=5)	3	2

Supplementary Table 2. Sample characteristics for the comparison of NULISAseq with other immunoassays

	Healthy controls (n=79)	Patients with disease (n=72)
Age in years (mean (sd)) *	46.7 (10.3)	64.0 (14.1)
Female (n (%))	34 (43.0%)	42 (58.3%)
Disease group (n (%))		
Cancer		32 (44.4%)
Inflammatory disease		21 (29.2%)
Kidney disease		4 (5.6%)
Metabolic disease		9 (12.5%)
Neurological disease		6 (8.3%)

* Age information was missing for one healthy male participant; this was imputed using the mean age for healthy males. Age information was missing for one male patient with a cancer diagnosis; this was imputed using the mean age of male patients with cancer.

Supplementary Table 3. Detectability of 92 shared targets of the NULISAseq 200-plex and Olink Explore 384 Inflammation panels in 79 samples from healthy controls and 72 samples from patients with different diseases

Panel	All samples (n=151)	Healthy controls (n=79)	Patients with disease (n=72)
	n (%) detectable* targets		
NULISAseq	89 (96.7%)	89 (96.7%)	89 (96.7%)
Olink Explore	85 (92.4%)	86 (93.5%)	85 (92.4%)
	detectability (%): mean (sd)/median [min, max]		
NULISAseq	95.9 (14.3)/100.0 [14.6, 100.0]	95.4 (15.8)/100.0 [3.8, 100.0]	96.4 (12.7)/100.0 [26.4, 100.0]
Olink Explore	91.6 (21.3)/100.0 [4.0, 100.0]	91.1 (22.0)/100.0 [1.3, 100.0]	92.2 (20.7)/100.0 [6.9, 100.0]

* A detectable target was defined as one for which the values were above the limit of detection for >50% of samples. The detectability was calculated as the percentage of samples in which the target signal was above the LOD. Source data are provided in Supplementary Data 2.

Supplementary Table 4. Sample characteristics for the characterization of the host response to SARS-CoV-2 infection with NULISAseq

	Healthy controls (n=16)	Patients with mild COVID-19 (n=9)
Age in years (mean (sd))	57.3 (13.6)	47.0 (19.4)
Female (n (%))	7 (43.8%)	6 (66.7%)
		Number of observations for each time period
t_{-1} (2-7 days before t_0*)		11
t_0		9
t_1 (2-7 days after t_0)		13
t_2 (8-20 days after t_0)		13

*t_0 was the time point at which the SARS-CoV-2 Nucleocapsid (N)protein level was maximum.

Supplementary Figures

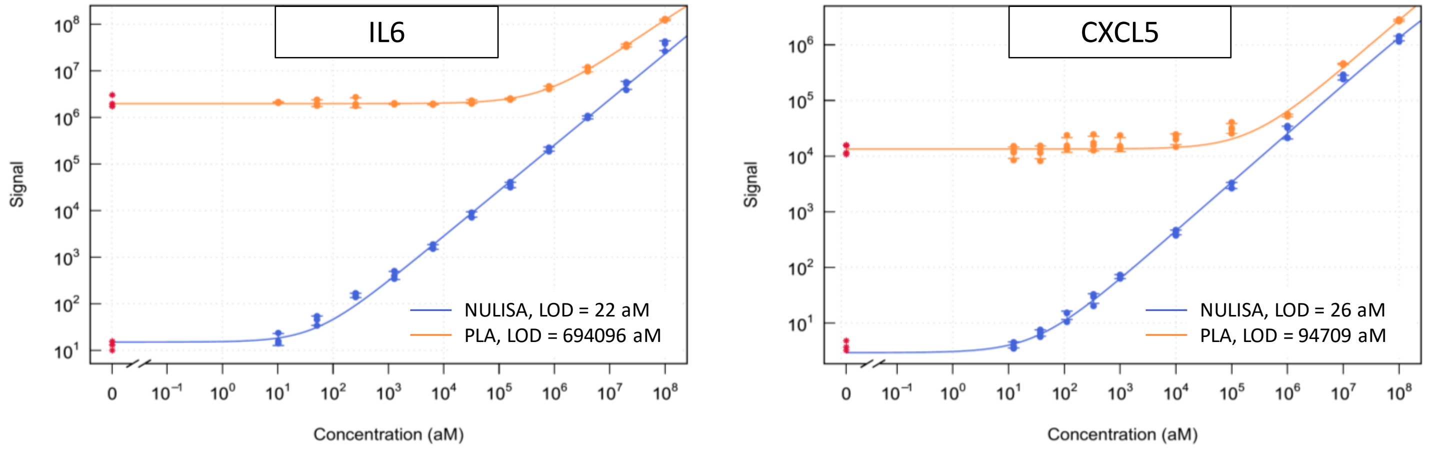

Supplementary Fig. 1. Comparison of standard curves generated from NULISA and PLA for IL6 and CXCL5. The same reagents including conjugated antibodies were used for NULISA and PLA assays. N=3. Source data are provided as a Source data file.

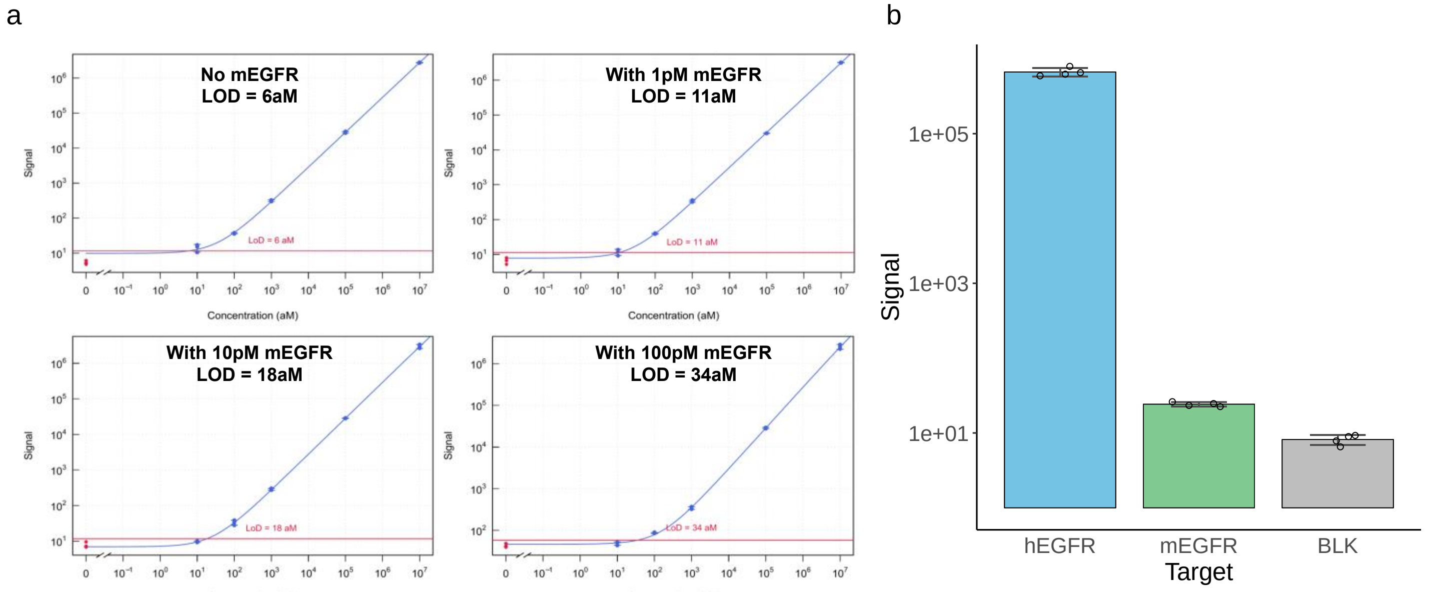

Supplementary Fig. 2. Specificity of the NULISA assay for human EGFR in the presence of mouse Egfr. **a**, Limit of detection (horizontal red line) for human EGFR measured in the presence of 0, 1, 10, and 100 pM mouse Egfr. N=3. **b**, NULISA signal of 10 pM human EGFR (hEGFR, teal), 10 pM mouse Egfr (mEGFR, green), and the blank control (BLK, gray). N=4. Source data are provided as a Source data file.

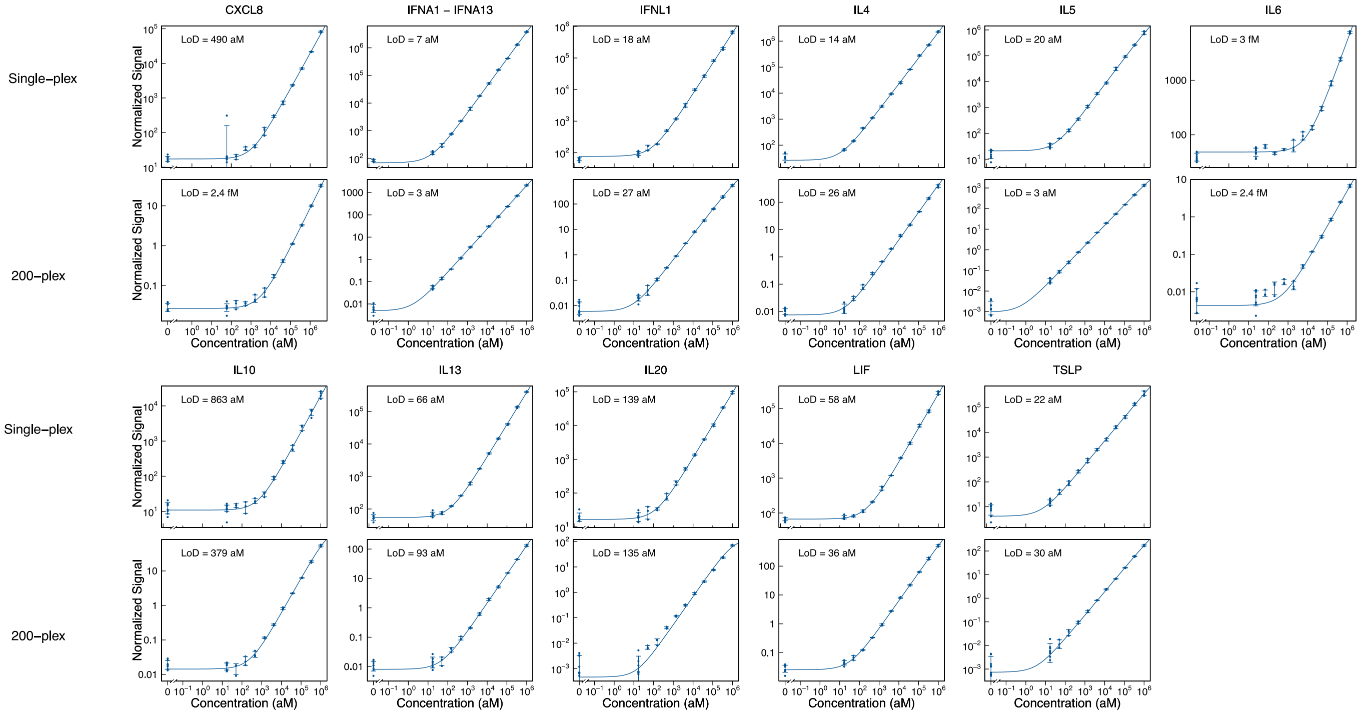

Supplementary Fig. 3. Comparison of standard curves generated from NULISAseq 200-plex and NULISA single-plex assays. Error bars represent mean +/- one standard deviation. N=3. Source data are provided as a Source data file.

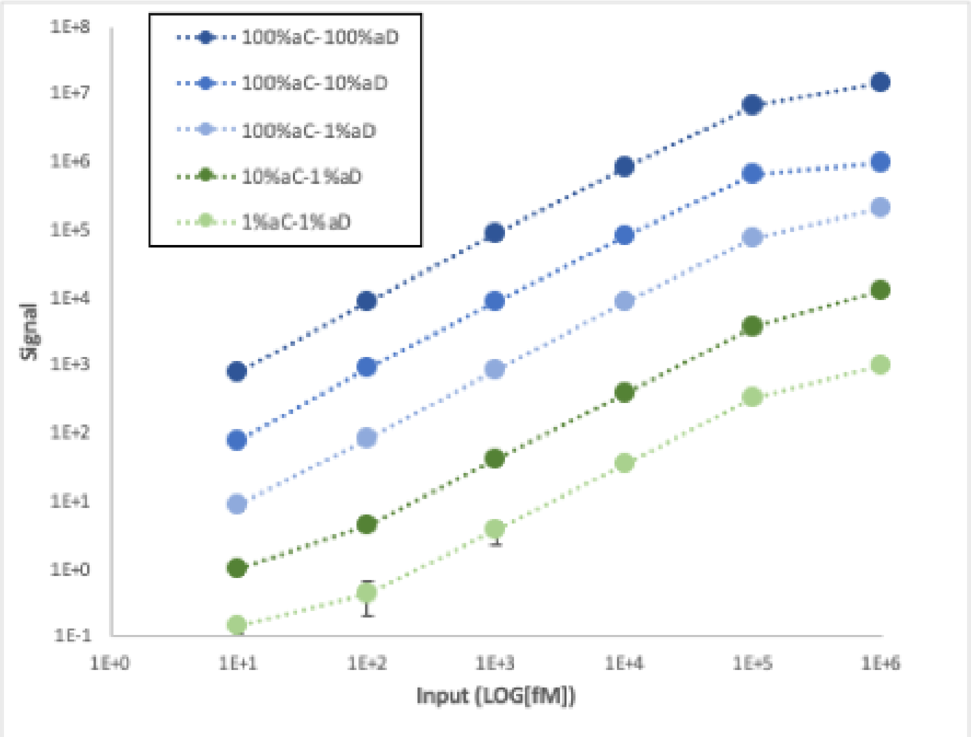

Supplementary Fig. 4. Signal tuning for multiplex NULISAseq assay. Standard curves of the IL4 assay were generated using different hot:cold antibody ratios. The total concentrations of both capture (aC) and detection (aD) antibodies after mixing were maintained the same for all conditions. The percentages of hot-capture and detection antibodies used to generate each curve are indicated. Six levels of protein calibrators were used to

assess the signal reduction in each condition when cold antibody was added. N=3. Source data are provided as a Source data file.

a

Target	Set 1 pools	Set 2 pools
CXCL13	1	13
IL18	1	22
IL5RA	1	38
CXCL1	1	39
MMP3	1	42
FAM3D	2	4
GDF15	2	6
OSM	2	8
CEACAM5	2	9
TNFRSF1A	2	28
MMP10	3	7
GFAP	3	10
CD274	3	17
ANGPT1	3	29
MICB	3	38
IL32	4	6
TREM1	4	20
CXCL3	4	32
IL7	4	33
CLEC4A	4	43
IL7R	5	2
IL18BP	5	4
TNFRSF11A	5	12
NGF	5	26
SPP1	5	36
CD40	6	5
IRAK4	6	7
TNFRSF4	6	14
TNFSF10	6	21
S100A9	6	24

… to pool #45

b

>50% homology to CEACAM5
CEACAM3
CEACAM6
CEACAM8
CEACAM1
CEACAM7
PSG9
PSG11
PSG2

c

>50% homology to IFNA1
IFNA8
IFNA10
IFNA21
IFNA5
IFNA14
IFNA17
IFNA7
IFNA6
IFNA4
IFNA16

d

>70% homology to any targets
LGALS9B
CCL3L1
CCL4L1
HPCAL4
IFNA5
CXCL6
FTMT
WNT7B
IFNL3
CD200R1L
CEACAM1
GZMH

e

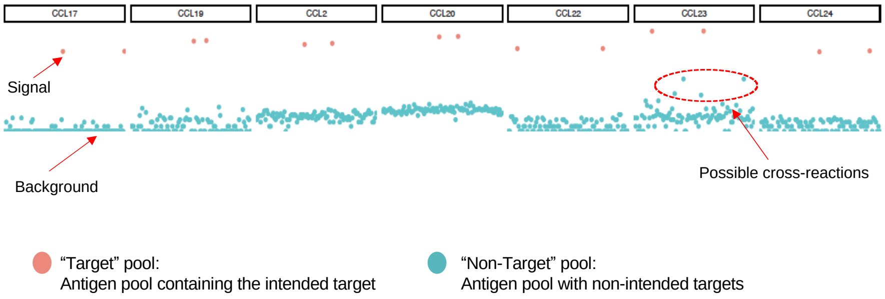

Cross Reactivity = (Max Signal in Non-Target Pools – Background)/(Mean Signal in Target Pools – Background) x 100

where Background = Median Signal in Non-Target Pools

***Each antigen was added at 20pM.**

Supplementary Fig. 5. Cross reaction testing with antigen pools. **a**) 198 antigens targeted by the 200plex assays were randomly partitioned into 45 pools with 4-5 antigens in each pool, and this was repeated to create a second set of 45 pools with the restriction that any two antigens were together only in one of the 90 pools. Three other antigen pools were also created to test cross reactivity to proteins not targeted by the 200plex panel: **b**) one pool of 8 proteins with >50% homology to CEACAM; **c**) a second pool of 10 proteins with >50% homology to IFNA1; **d)** a third pool of 12 proteins with >70% homology to any target in the 200plex panel. **e**) Each protein, either target analyte or nontarget, was at 20 pM concentration in each pool. These antigen pools were treated as individual samples and analyzed with the NULISAseq 200plex assay. Examples of the resulting NULISAseq data are shown, plotting the read count in log2 scale for the target of interest in the y-axis and antigen pools in the x-axis. Red dots denote counts from the two target-containing pools, and blue dots denote signals from pools not containing the target. The median read count from all nontarget pools was calculated as the background. The cross-reactivity of each assay for its target was calculated using the formula shown.

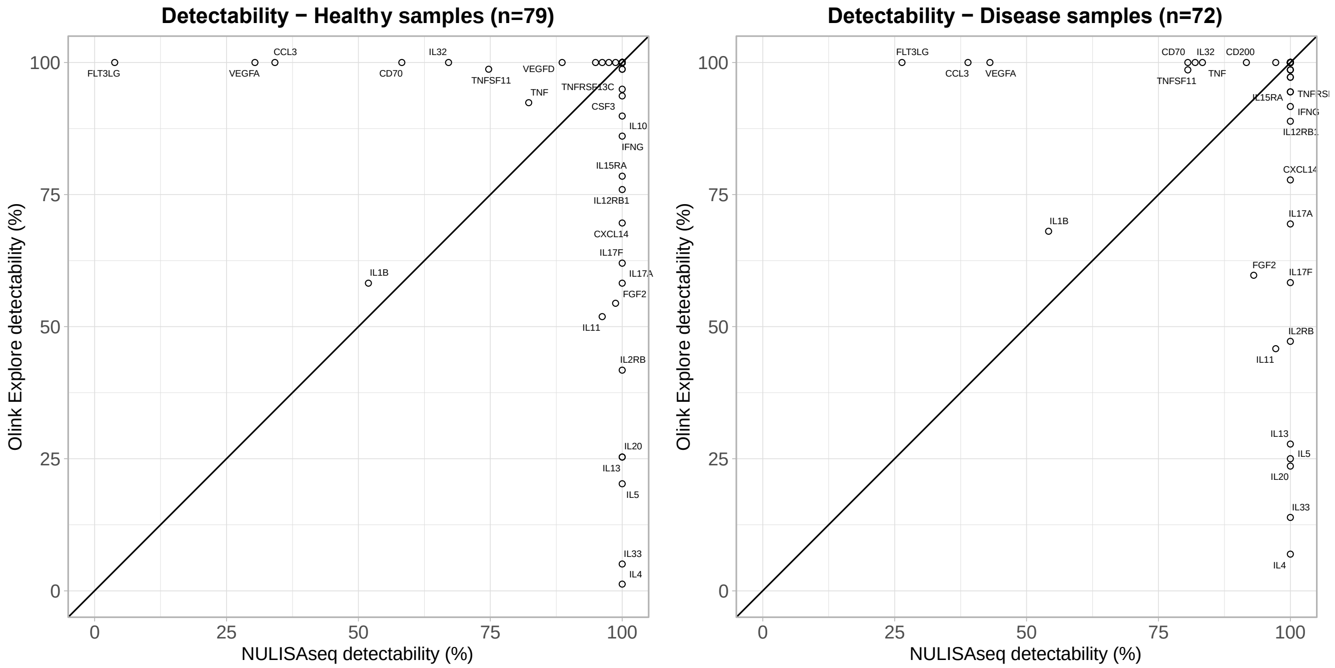

Supplementary Fig. 6. Detectability of 92 common targets shared between the NULISAseq 200-plex and the Olink Explore 384 Inflammation Panel. Detectability was calculated for each target as the percentage of samples with results above the LOD using 79 samples from healthy controls (left) or 72 samples from patients with different diseases (right). Source data are provided in Supplementary Data 2.

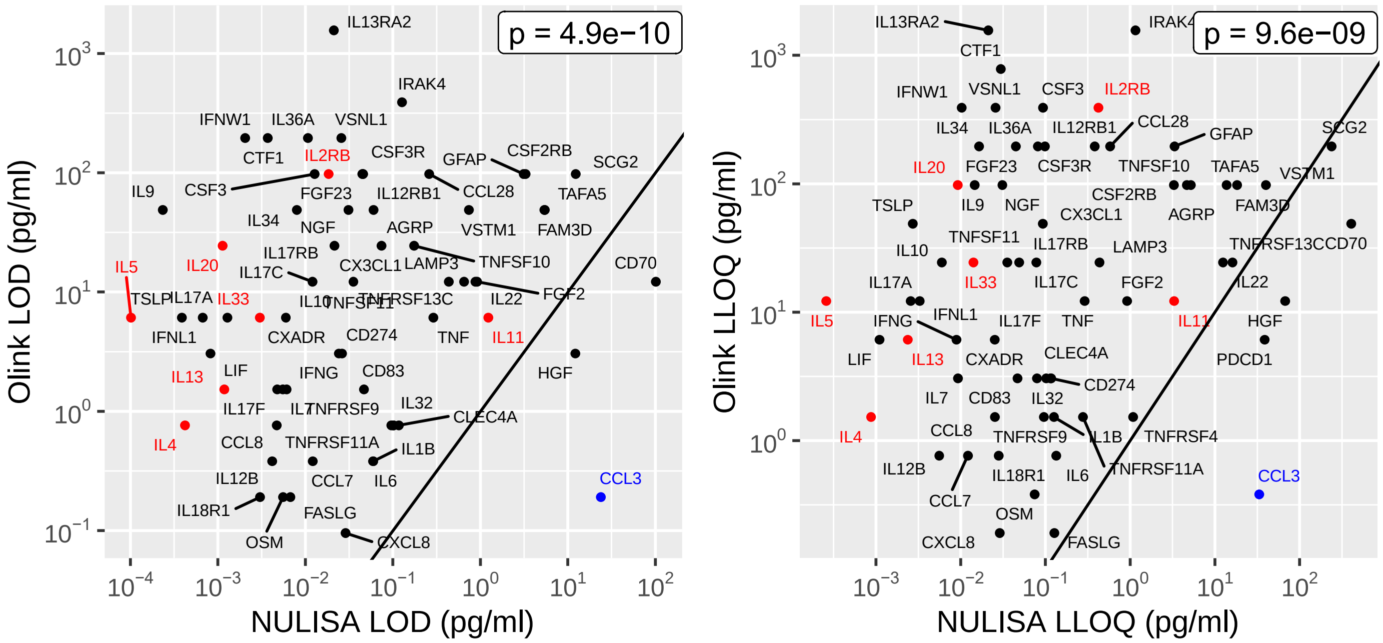

Supplementary Fig. 7. Comparison of LOD and LLOQ between NULISA and Olink Explore 3072. NULISA LODs and LLOQs were from Supplemental Data A, and Olink LODs and LLOQs were from Olink's Explore 3072 validation datasheet (Document download center – Olink). Excluding assays that were tuned with hot and cold mixing in NULISAseq 200plex and those requiring sample dilution in Olink Explore, 74 shared targets between the two platforms were identified. NULISA and Olink LOD and LLOQ data for these targets were plotted. P values were two-sided from nonparametric paired Wilcoxon test. The diagonal line indicates identity. Targets were highlighted according to detectability differences shown in Supplementary Fig. 6: in red if Olink detectability (Supplementary Fig. 6) were <50% whereas NULISAseq detectability were >= 50%, and in blue if Olink detectability were >=50% whereas NULISAseq detectability were <50%. Source data are provided in Supplementary Data 3.

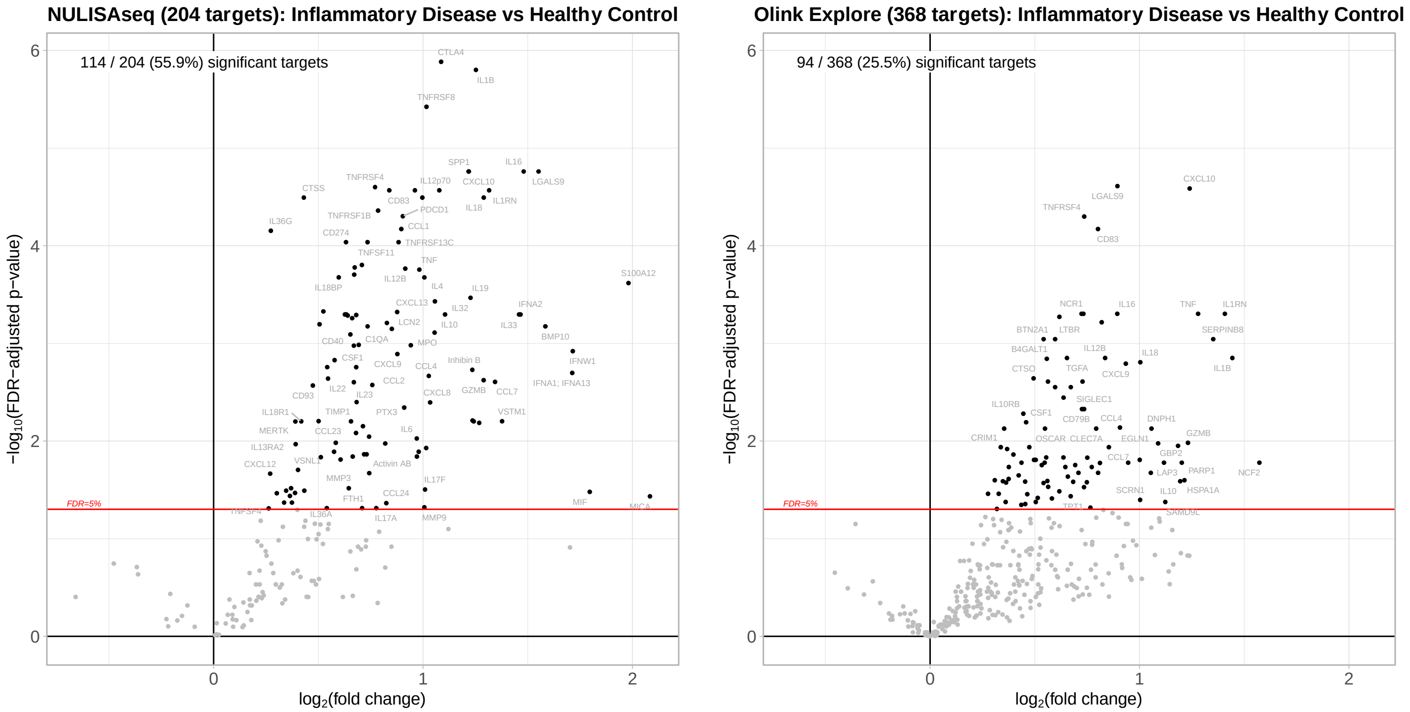

Supplementary Fig. 8. Differential expression analysis comparing n=21 patients with inflammatory diseases and n=79 healthy controls for the 204 targets in the NULISAseq 200-plex Inflammation Panel (left) and 368 targets in the Olink Explore 384 Inflammation Panel (right). Volcano plots show –log10 FDR-adjusted p value versus estimated log2-fold changes for the inflammatory disease group, relative to healthy control, from linear models adjusted for age, sex, and plate. Source data are provided as a Source data file.

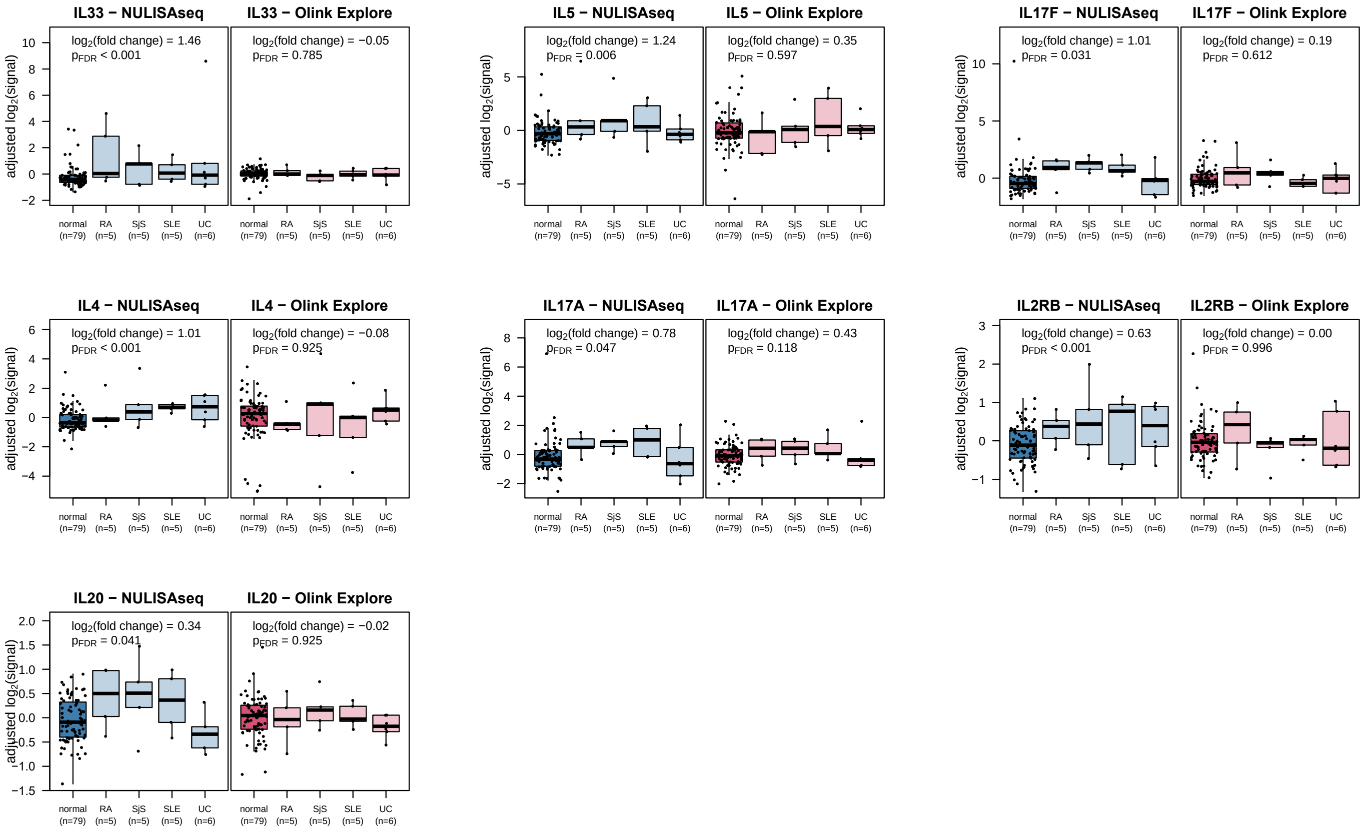

Supplementary Fig. 9. Selected low-abundance targets associated with autoimmune diseases detected by NULISAseq but not by Olink Explore. Boxplots show the distributions of residuals (after adjusting for age, sex, and plate) for healthy controls versus inflammatory disease subgroups. FDR-adjusted p values were obtained based on the disease status (inflammatory disease, n=21, versus healthy control, n=79) coefficient from linear models (adjusted for age, sex, and plate) used in the differential expression analysis of 92 shared NULISAseq and Olink targets. Boxplots show lines at median; boxes indicate interquartile range; whiskers show values extending from interquartile range to up to 1.5 times the interquartile range. RA: rheumatoid arthritis (n=5), SjS: Sjögren’s syndrome (n=5), SLE: systemic lupus erythematosus (n=5), UC: ulcerative colitis (n=6). Source data are provided as a Source data file.

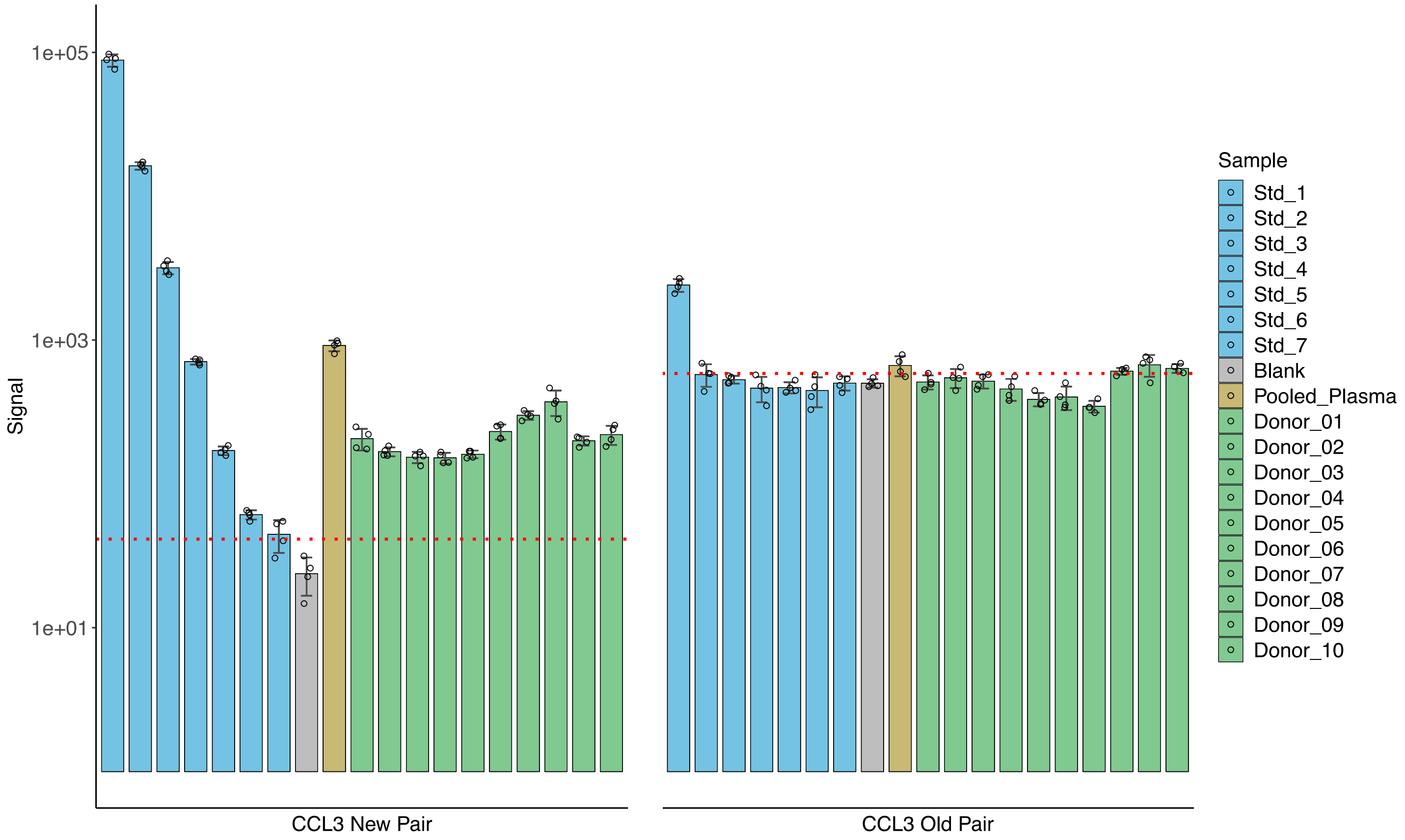

Supplementary Fig. 10. Improved CCL3 assay with a different antibody pair. Bar graphs show the signal difference between CCL3 NULISA assays using new (left) and old (right) pair of antibodies. Different sample types are marked with colors: blue – standards (10-fold serial dilution from 10pM), gray – blank, olive – pooled plasma, green – individual plasma samples. Red dotted lines indicate the LOD for each pair of antibodies. N=4. Source data are provided as a Source data file.
