# Supplementary material for: NULISA: a proteomic liquid biopsy platform with attomolar sensitivity and high multiplexing
Source: Nat Commun. 2023 Nov 9;14:7238. doi: 10.1038/s41467-023-42834-x (PMC10636041; doi:10.1038/s41467-023-42834-x)
Supplement: Supplementary file 3 — Description of Additional Supplementary Files Document [file 41467_2023_42834_MOESM3_ESM.pdf]

Description of Additional Supplementary files

Supplementary Data 1. NULISAseq 200-plex Inflammation Panel and performance summary (precision, LOD, dynamic range, detectability, and specificity). The column “Hot/cold signal tuning” indicates whether the assay sensitivity for the target was tuned down with hot/cold antibody mixing, which would result in higher LODs but should maintain sample detectability.

Supplementary Data 2. Detailed results obtained by comparing NULISAseq and Olink Explore. Columns with names ending with “_log2_fold_change”, “_pval” and “_pval_FDR” list the regression coefficients (log2-fold change), unadjusted p values and FDR-adjusted p values, respectively, from linear models assessing differential expression between 79 samples from healthy controls and 21 samples from patients with inflammatory disease, adjusted for age, sex, and plate. Columns with names containing “detect” list target detectability, based on the same samples from 79 healthy controls and an expanded set of 72 samples from patients with various diseases, and those with names containing “CV” list intra- and interplate CV%.

Supplementary Data 3. This file has two worksheets. Sheet 1, comparison of LOD and LLOQ between NULISA and Olink Explore 3072. NULISA LODs and LLOQs were from Supplemental Data A, and Olink LODs and LLOQs were from Olink’s Explore 3072 validation datasheet (Document download center – Olink). Excluding assays that were tuned with hot and cold mixing in NULISAseq 200-plex and those requiring sample dilution in Olink Explore, 74 shared targets between the two platforms were identified. The LOD and LLOQ ratio of Olink to NULISA were calculated for each target. The median LOD, LLOQ and LOD and LLOQ ratios across all targets were summarized at the bottom row. Sheet 2, comparison of LOD, LLOQ and detectability for the 45 targets shared between NULISAseq 200-plex and Olink Explore Inflammation Panel.

Supplementary Data 4. Detailed differential expression analysis results for the comparison of samples from patients with mild COVID-19 and healthy donors. Sheet 1 (limma_analysis) lists the full results with the log2(fold change) and FDR-adjusted p values; sheet 2 (limma_results_top20) lists the top 20 significantly different targets ranked by maximum fold change; and sheet 3 (Enrichment) lists the metascape pathway analysis results based on all significantly different targets.

Supplementary Data 5. List of antibodies used in the NULISAseq 200-plex inflammation panel
