# Supplementary material for: NULISA: a proteomic liquid biopsy platform with attomolar sensitivity and high multiplexing
Source: Nat Commun. 2023 Nov 9;14:7238. doi: 10.1038/s41467-023-42834-x (PMC10636041; doi:10.1038/s41467-023-42834-x)
Supplement: Supplementary file 9 — Reporting Summary [file 41467_2023_42834_MOESM9_ESM.pdf]

nature portfolio

Corresponding author(s): Xiao-Jun Ma, Yuling Luo

Last updated by author(s): Oct 6, 2023

Reporting Summary

Nature Portfolio wishes to improve the reproducibility of the work that we publish. This form provides structure for consistency and transparency in reporting. For further information on Nature Portfolio policies, see our Editorial Policies and the Editorial Policy Checklist.

Statistics

For all statistical analyses, confirm that the following items are present in the figure legend, table legend, main text, or Methods section.

n/a	Confirmed	
☐	☒	The exact sample size (*n*) for each experimental group/condition, given as a discrete number and unit of measurement
☐	☒	A statement on whether measurements were taken from distinct samples or whether the same sample was measured repeatedly
☐	☒	The statistical test(s) used AND whether they are one- or two-sided<br>*Only common tests should be described solely by name; describe more complex techniques in the Methods section.*
☐	☒	A description of all covariates tested
☐	☒	A description of any assumptions or corrections, such as tests of normality and adjustment for multiple comparisons
☐	☒	A full description of the statistical parameters including central tendency (e.g. means) or other basic estimates (e.g. regression coefficient) AND variation (e.g. standard deviation) or associated estimates of uncertainty (e.g. confidence intervals)
☐	☒	For null hypothesis testing, the test statistic (e.g. *F*, *t*, *r*) with confidence intervals, effect sizes, degrees of freedom and *P* value noted<br>*Give P values as exact values whenever suitable.*
☒	☐	For Bayesian analysis, information on the choice of priors and Markov chain Monte Carlo settings
☒	☐	For hierarchical and complex designs, identification of the appropriate level for tests and full reporting of outcomes
☐	☒	Estimates of effect sizes (e.g. Cohen's *d*, Pearson's *r*), indicating how they were calculated

Our web collection on statistics for biologists contains articles on many of the points above.

Software and code

Policy information about availability of computer code

Data collection	qPCR data was collected using BioRad CFX Maestro Manager 2.2 (v4.2.008.0222), NGS data was collected using Illumina BaseSpace Sequence Hub (v7.9.0) and ARGO Command Center (v0.2.0.0).
Data analysis	All data analyses were performed using R software (v.4.2.0, R Core Team, 2021). An open-source R package (GPLv3) for analyzing NULISAseq data is available at https://github.com/Alamar-Biosciences/NULISAseqR (v1.0).

For manuscripts utilizing custom algorithms or software that are central to the research but not yet described in published literature, software must be made available to editors and reviewers. We strongly encourage code deposition in a community repository (e.g. GitHub). See the Nature Portfolio guidelines for submitting code & software for further information.

Data

Policy information about availability of data

All manuscripts must include a data availability statement. This statement should provide the following information, where applicable:

- Accession codes, unique identifiers, or web links for publicly available datasets
- A description of any restrictions on data availability
- For clinical datasets or third party data, please ensure that the statement adheres to our policy

All data generated and analyzed in this study are included in this manuscript, its supplementary information, or have been made available in public repositories. Reference concentrations of blood proteins are available at Human Protein Atlas (https://www.proteinatlas.org/humanproteome/blood+protein). The NULISAseq

200-plex Inflammation Panel data and Olink Explore Inflammation Panel data generated in this study have been deposited in the National Center for Biotechnology Information (NCBI) Gene Expression Omnibus (GEO) database under accession code GSE241717 [https://www.ncbi.nlm.nih.gov/geo/query/acc.cgi?acc=GSE241717].

Source data for Figures 2b, 2c, 3c, and 4a are provided with the paper. Figures 3a and 3b were generated using data in Supplementary Data 1. Figure 3d was generated using data in the Alamar_NULISAseq_Detectability_NPQ.csv file available under accession GSM7734322 [https://www.ncbi.nlm.nih.gov/geo/query/acc.cgi?acc=GSM7734322]. Figures 4b, 4c, 4d, and 4e were generated using data in Supplementary Data 2. Figures 5a and 5b were generated using data in the Alamar_NULISAseq_COVID_NPQ.csv file available under accession GSM7734324 [https://www.ncbi.nlm.nih.gov/geo/query/acc.cgi?acc=GSM7734324].

Research involving human participants, their data, or biological material

Policy information about studies with human participants or human data. See also policy information about sex, gender (identity/presentation), and sexual orientation and race, ethnicity and racism.

Reporting on sex and gender	Sex was included as a covariate in differential expression models comparing samples from patients with inflammatory disease (n=21, 18 female) and healthy controls (n=72, 42 female), and in models assessing the differences between mild COVID-19 (n=9, 6 female) and healthy control (n=16, 7 female). Because the impact of sex on protein abundance was not of primary interest in this study, we do not report statistics for the sex covariate. However, the data, including age and sex, are available in the files uploaded to National Center for Biological Information (NCBI) Gene Expression Omnibus (GEO) repository with accession number GSE241717.
Reporting on race, ethnicity, or other socially relevant groupings	Age and sex were the only potential confounding variables considered in the linear models. We did not have information about other socially relevant groupings for these data, unfortunately. We may not have had the statistical power to include more potential confounders, such as race, ethnicity, or other socially relevant groupings.
Population characteristics	Patient demographics and characteristics are summarized in Suplementary Tables 1, 2 and 4.
Recruitment	This cohort has been described previously: Jalota, A., Hershberger, C. E., Patel, M. S., Mian, A., Khademi, G., Rotroff, D. M., Hill, B. T., & Gupta, N. (n.d.). Host metabolome predicts the severity and onset of acute toxicities induced by CAR T-cell therapy Clinical trial registration information (if any): Host metabolome predicts the severity and onset of acute toxicities induced by CAR T-cell 1 therapy 2 3. https://doi.org/10.1182/bloodadvances.2022007456/2007084/bloodadvances.2022007456.pdf
Ethics oversight	The COVID-19 study was approved by th University Hospital of Bonn institutional research ethics board (ethics vote 468/20).

Note that full information on the approval of the study protocol must also be provided in the manuscript.

Field-specific reporting

Please select the one below that is the best fit for your research. If you are not sure, read the appropriate sections before making your selection.

☒ Life sciences ☐ Behavioural & social sciences ☐ Ecological, evolutionary & environmental sciences

For a reference copy of the document with all sections, see nature.com/documents/nr-reporting-summary-flat.pdf

Life sciences study design

All studies must disclose on these points even when the disclosure is negative.

Sample size	no sample size calculation was performed. Study sample sizes were based on sample availability.
Data exclusions	two data points were excluded in the COVID-19 data analysis of differential expression because no N protein data were available
Replication	NULISAseq data for sample detectability and COVID-19 were not replicated as each sample was measured once. This was because each clinical sample was unique and repeated samples from the same patients were not available. A technical replicate was included on each run to assess technical variation.
Randomization	Since we aimed to study blood proteomic inflammatory profiles in disease (inflammatory diseases, COVID-19) versus healthy individuals, no randomization of group assignment was done, but rather a case-control type study design was employed. Differences in age and sex between disease and healthy groups were accounted for by using these variables as covariates in linear models. Randomization was applied when assigning samples to the different assay plates, to prevent potential confounding of the biological factors of interest based on run-run variation.
Blinding	Given the case-control nature of the study, investigators were not blinded during collection or analysis. The lack of blinding is not expected to have impacted the data collection (blood samples) or analysis. Disease status was necessary for investigators to know for the purposes of randomization to assay plates and analysis of the biological factors of interest (e.g., impact of disease status on protein abundance).

Reporting for specific materials, systems and methods

We require information from authors about some types of materials, experimental systems and methods used in many studies. Here, indicate whether each material, system or method listed is relevant to your study. If you are not sure if a list item applies to your research, read the appropriate section before selecting a response.

Materials & experimental systems

n/a	Involved in the study
☐	☒ Antibodies
☒	☐ Eukaryotic cell lines
☒	☐ Palaeontology and archaeology
☒	☐ Animals and other organisms
☒	☐ Clinical data
☒	☐ Dual use research of concern
☒	☐ Plants

Methods

n/a	Involved in the study
☒	☐ ChIP-seq
☒	☐ Flow cytometry
☒	☐ MRI-based neuroimaging

Antibodies

Antibodies used

All antibodies used in this study were primary antibodies. No secondary antibodies were used. The antibodies used in the NULISAseq 200-plex Inflammation Panel were listed in Supplementary Data 5 and shown below, containing target name, unique Alamar antibody ID, host species and clonality. This will allow researchers who wish to replicate the work to use the same reagents. In addition, antibodies were conjugated to DNA and purified by Alamar.

,,Capture antibody,,,Detection antibody,,
Target,Uniprot,Alamar Antibody ID,Host Species,Clonality,Alamar Antibody ID,Host Species,Clonality
Activin AB,P08476,cAb3150,Mouse,Monoclonal,cAb2779,Mouse,Monoclonal
AGER,Q15109,cAb3592,Rabbit,Monoclonal,cAb3562,Rabbit,Monoclonal
AGRP,O00253,cAb3661,Goat,Polyclonal,cAb1304,Rabbit,Monoclonal
ANGPT1,Q15389,cAb2443,Goat,Polyclonal,cAb2444,Mouse,Monoclonal
ANXA1,P04083,cAb4435,Mouse,Monoclonal,cAb4436,Mouse,Monoclonal
BDNF,P23560,cAb3338,Rabbit,Polyclonal,cAb3915,Mouse,Monoclonal
BMP10,O95393,cAb3925,Mouse ,Monoclonal,cAb2551,Mouse,Monoclonal
BMP7,P18075,cAb2059,Mouse,Monoclonal,cAb1569,Rabbit,Monoclonal
C1QA,P02745,cAb1503,Rabbit,Monoclonal,cAb1504,Rabbit,Monoclonal
CCL1,P22362,cAb4340,Mouse,Monoclonal,cAb4343,Goat,Polyclonal
CCL13,Q99616,cAb486,Rabbit,Monoclonal,cAb481,Rabbit,Monoclonal
CCL14,Q16627,cAb1581,Rabbit,Monoclonal,cAb1582,Rabbit,Monoclonal
CCL15,Q16663,cAb2851,Mouse,Monoclonal,cAb4255,Goat,Polyclonal
CCL16,O15467,cAb1586,Rabbit,Monoclonal,cAb1585,Rabbit,Monoclonal
CCL17,Q92583,cAb2012,Mouse,Monoclonal,cAb3662,Rabbit,Monoclonal
CCL19,Q99731,cAb1587,Rabbit,Monoclonal,cAb1588,Rabbit,Monoclonal
CCL2,P13500,cAb3392,Mouse,Monoclonal,cAb3625,Mouse,Monoclonal
CCL20,P78556,cAb3397,Rabbit,Monoclonal,cAb3629,Rabbit,Monoclonal
CCL22,O00626,cAb1589,Rabbit,Monoclonal,cAb1590,Rabbit,Monoclonal
CCL23,P55773,cAb838,Rabbit,Monoclonal,cAb839,Rabbit,Monoclonal
CCL24,O00175,cAb577,Rabbit,Monoclonal,cAb576,Rabbit,Monoclonal
CCL25,O15444,cAb634,Rabbit,Monoclonal,cAb635,Rabbit,Monoclonal
CCL28,Q9NRJ3,cAb2036,Goat,Polyclonal,cAb2014,Mouse,Monoclonal
CCL3,P10147,cAb408,Rabbit,Monoclonal,cAb440,Rabbit,Monoclonal
CCL4,P13236,cAb2929,Rabbit,Monoclonal,cAb2930,Rabbit,Monoclonal
CCL5,P13501,cAb3700,Rabbit,Monoclonal,cAb3701,Rabbit,Monoclonal
CCL7,P80098,cAb2046,Goat,Polyclonal,cAb1441,Rabbit,Monoclonal
CCL8,P80075,cAb528,Rabbit,Monoclonal,cAb410,Rabbit,Monoclonal
CD200,P41217,cAb1597,Rabbit,Monoclonal,cAb1598,Rabbit,Monoclonal
CD200R1,Q8TD46,cAb1599,Rabbit,Monoclonal,cAb1600,Rabbit,Monoclonal
CD274,Q9NZQ7,cAb674,Rabbit,Monoclonal,cAb675,Rabbit,Monoclonal
CD276,Q5ZPR3,cAb3703,Rabbit,Monoclonal,cAb3702,Rabbit,Monoclonal
CD40,P25942,cAb4341,Rabbit,Monoclonal,cAb4344,Rabbit,Monoclonal
CD70,P32970,cAb413,Rabbit,Monoclonal,cAb412,Rabbit,Monoclonal
CD83,Q01151,cAb729,Rabbit,Monoclonal,cAb730,Rabbit,Monoclonal
CD93,Q9NPY3,cAb1617,Rabbit,Monoclonal,cAb1618,Rabbit,Monoclonal
CEACAM5,P06731,cAb3394,Mouse,Monoclonal,cAb3627,Mouse,Monoclonal
CHI3L1,P36222,cAb3418,Rabbit,Monoclonal,cAb3649,Rabbit,Monoclonal
CLCF1,Q9UBD9,cAb4071,Mouse ,Monoclonal,cAb4070,Goat,Polyclonal
CLEC4A,Q9UMR7,cAb1875,Mouse,Monoclonal,cAb1876,Mouse,Monoclonal
CSF1,P09603,cAb3395,Rabbit,Monoclonal,cAb911,Rabbit,Monoclonal
CSF2RB,P32927,cAb4437,Human,Monoclonal,cAb3037,Goat,Monoclonal
CSF3,P09919,cAb913,Rabbit,Monoclonal,cAb914,Rabbit,Monoclonal
CSF3R,Q99062,cAb3028,Rabbit,Monoclonal,cAb3025,Mouse,Monoclonal
CST7,O76096,cAb3230,Rabbit,Polyclonal,cAb3231,Mouse,Monoclonal
CTF1,Q16619,cAb2331,Rabbit,Monoclonal,cAb2332,Rabbit,Monoclonal
CTLA4,P16410,cAb2265,Mouse,Monoclonal,cAb4226,Rabbit,Monoclonal
CTSS,P25774,cAb4196,Rabbit,Polyclonal,cAb3446,Goat,Polyclonal

CX3CL1,P78423,cAb4203,Rabbit,Polyclonal,cAb4203,Rabbit,Polyclonal
CXADR,P78310,cAb847,Rabbit,Monoclonal,cAb846,Rabbit,Monoclonal
CXCL1,P09341,cAb1626,Rabbit,Monoclonal,cAb1625,Rabbit,Monoclonal
CXCL10,P02778,cAb1364,Rabbit,Monoclonal,cAb1365,Rabbit,Monoclonal
CXCL12,P48061,cAb2049,Goat,Polyclonal,cAb3420,Rabbit,Polyclonal
CXCL13,O43927,cAb3401,Rabbit,Monoclonal,cAb3633,Rabbit,Monoclonal
CXCL14,O95715,cAb3452,Rat,Monoclonal,cAb4373,Goat,Polyclonal
CXCL2,P19875,cAb1627,Rabbit,Monoclonal,cAb1628,Rabbit,Monoclonal
CXCL3,P19876,cAb3403,Rabbit,Monoclonal,cAb3635,Rabbit,Monoclonal
CXCL5,P42830,cAb142,Mouse,Monoclonal,cAb143,Mouse,Monoclonal
CXCL8,P10145,cAb068,Mouse,Monoclonal,cAb4595,Mouse,Monoclonal
CXCL9,Q07325,cAb4342,Rabbit,Monoclonal,cAb3739,Rabbit,Monoclonal
EGF,P01133,cAb426,Rabbit,Monoclonal,cAb409,Rabbit,Monoclonal
EPO,P01588,cAb1634,Rabbit,Monoclonal,cAb1633,Rabbit,Monoclonal
FAM3D,Q96BQ1,cAb2343,Mouse,Monoclonal,cAb2344,Goat,Monoclonal
FASLG,P48023,cAb2019,Mouse,Monoclonal,cAb3663,Goat,Monoclonal
FGF19,O95750,cAb1644,Rabbit,Monoclonal,cAb1643,Rabbit,Monoclonal
FGF2,P09038,cAb3406,Rabbit,Monoclonal,cAb3637,Rabbit,Monoclonal
FGF21,Q9NSA1,cAb3763,Rabbit,Monoclonal,cAb3762,Rabbit,Monoclonal
FGF23,Q9GZV9,cAb2069,Mouse,Monoclonal,cAb2092,Goat,Polyclonal
FLT1,P17948,cAb1645,Rabbit,Monoclonal,cAb1646,Rabbit,Monoclonal
FLT3LG,P49771,cAb1647,Rabbit,Monoclonal,cAb1648,Rabbit,Monoclonal
FTH1,P02794,cAb2182,Mouse,Monoclonal,cAb2183,Mouse,Monoclonal
GDF15,Q99988,cAb3770,Mouse ,Monoclonal,cAb3771,Mouse,Monoclonal
GDF2,Q9UK05,cAb3405,Rabbit,Monoclonal,cAb3636,Rabbit,Monoclonal
GFAP,P14136,cAb3408,Rabbit,Monoclonal,cAb3639,Rabbit,Monoclonal
GRN,P28799,cAb3404,Rabbit,Monoclonal,cAb649,Rabbit,Monoclonal
GZMB,P10144,cAb3471,Rabbit,Monoclonal,cAb3472,Human,Monoclonal
HGF,P14210,cAb806,Rabbit,Monoclonal,cAb923,Rabbit,Monoclonal
IFNA1_IFNA13,P01562,cAb496,Rabbit,Monoclonal,cAb497,Rabbit,Monoclonal
IFNA2,P01563,cAb2364,Mouse,Monoclonal,cAb4397,Mouse,Monoclonal
IFNB1,P01574,cAb1664,Rabbit,Monoclonal,cAb1663,Rabbit,Monoclonal
IFNG,P01579,cAb2291,Mouse,Monoclonal,cAb2073,Mouse,Monoclonal
IFNL1,Q8IU54,cAb2528,Rabbit,Monoclonal,cAb2523,Mouse,Monoclonal
IFNW1,P05000,cAb1462,Rabbit,Monoclonal,cAb3658,Mouse,Monoclonal
IL-12p70,P29459,cAb1810,Rabbit,Monoclonal,cAb3085,Rabbit,Monoclonal
IL-23,Q9NPF7,cAb1810,Rabbit,Monoclonal,cAb3092,Rabbit,Monoclonal
IL-27,Q8NEV9,cAb1810,Rabbit,Monoclonal,cAb454,Rabbit,Monoclonal
IL-35,P29459,cAb404,Rat,Monoclonal,cAb3085,Rabbit,Monoclonal
IL-39,Q9NPF7,cAb752,Rabbit,Monoclonal,cAb3092,Rabbit,Monoclonal
IL10,P22301,cAb2042,Goat,Polyclonal,cAb405,Rat,Monoclonal
IL10RB,Q08334,cAb450,Rabbit,Monoclonal,cAb753,Rabbit,Monoclonal
IL11,P20809,cAb450,Rabbit,Monoclonal,cAb3656,Goat,Monoclonal
IL12B,P29460,cAb450,Rabbit,Monoclonal,cAb536,Rabbit,Monoclonal
IL12RB1,P42701,cAb1324,Rabbit,Monoclonal,cAb1325,Rabbit,Monoclonal
IL13,P35225,cAb1520,Rabbit,Monoclonal,cAb1521,Rabbit,Monoclonal
IL13RA2,Q14627,cAb3398,Rabbit,Monoclonal,cAb3630,Rabbit,Monoclonal
IL15RA,Q13261,cAb3399,Rabbit,Monoclonal,cAb3631,Rabbit,Monoclonal
IL16,Q14005,cAb652,Rabbit,Monoclonal,cAb4884,Goat,Monoclonal
IL17A,Q16552,cAb4193,Mouse ,Monoclonal,cAb3489,Mouse,Monoclonal
IL17B,Q9UHF5,cAb3400,Rabbit,Monoclonal,cAb3632,Rabbit,Monoclonal
IL17C,Q9P0M4,cAb1669,Rabbit,Monoclonal,cAb1670,Rabbit,Monoclonal
IL17F,Q96PD4,cAb1796,Rabbit,Monoclonal,cAb1797,Rabbit,Monoclonal
IL17RB,Q9NRM6,cAb754,Rabbit,Monoclonal,cAb755,Rabbit,Monoclonal
IL18,Q14116,cAb3390,Rabbit,Monoclonal,cAb3797,Rabbit,Monoclonal
IL18BP,O95998,cAb1674,Rabbit,Monoclonal,cAb3493,Goat,Polyclonal
IL18R1,Q13478,cAb1921,Rabbit,Monoclonal,cAb1922,Mouse,Monoclonal
IL19,Q9UHD0,cAb1150,Rabbit,Monoclonal,cAb1463,Rabbit,Monoclonal
IL1B,P01584,cAb4557,Mouse,Monoclonal,cAb4905,Goat,Monoclonal
IL1R2,P27930,cAb809,Rabbit,Monoclonal,cAb810,Rabbit,Monoclonal
IL1RL1,Q01638,cAb3800,Rabbit,Monoclonal,cAb4264,Rabbit,Monoclonal
IL1RN,P18510,cAb541,Rabbit,Monoclonal,cAb542,Rabbit,Monoclonal
IL20,Q9NYY1,cAb423,Rabbit,Monoclonal,cAb543,Rabbit,Monoclonal
IL22,Q9GZX6,cAb3499,Mouse,Monoclonal,cAb3498,Mouse,Monoclonal
IL2RA,P01589,cAb1678,Rabbit,Monoclonal,cAb1677,Rabbit,Monoclonal
IL2RB,P14784,cAb1679,Rabbit,Monoclonal,cAb1680,Rabbit,Monoclonal
IL32,P24001,cAb1465,Rabbit,Monoclonal,cAb3517,Goat,Polyclonal
IL33,O95760,cAb4293,Mouse ,Monoclonal,cAb4263,Goat,Monoclonal
IL34,Q6ZMJ4,cAb3402,Rabbit,Monoclonal,cAb3634,Rabbit,Monoclonal
IL36A,Q9UHA7,cAb1466,Rabbit,Monoclonal,cAb1467,Rabbit,Monoclonal
IL36G,Q9NZH8,cAb3527,Goat,Polyclonal,cAb3268,Mouse,Monoclonal
IL3RA,P26951,cAb1328,Rabbit,Monoclonal,cAb1329,Rabbit,Monoclonal
IL4,P05112,cAb3802,Mouse ,Monoclonal,cAb4207,Rat,Monoclonal
IL4R,P24394,cAb3905,Rabbit,Monoclonal,cAb3906,Rabbit,Monoclonal
IL5,P05113,cAb609,Rabbit,Monoclonal,cAb4538,Rabbit,Monoclonal
IL5RA,Q01344,cAb1684,Rabbit,Monoclonal,cAb1683,Rabbit,Monoclonal

IL6,P05231,cAb1798,Rabbit,Monoclonal,cAb5144,Rabbit,Monoclonal
IL6R,P08887,cAb1686,Rabbit,Monoclonal,cAb1685,Rabbit,Monoclonal
IL7,P13232,cAb425,Rabbit,Monoclonal,cAb547,Rabbit,Monoclonal
IL7R,P16871,cAb1689,Rabbit,Monoclonal,cAb1690,Rabbit,Monoclonal
IL9,P15248,cAb2128,Mouse,Monoclonal,cAb455,Rabbit,Monoclonal
INHBA,P08476,cAb3150,Mouse,Monoclonal,cAb3150,Mouse,Monoclonal
INHBB,P09529,cAb2779,Mouse,Monoclonal,cAb2779,Mouse,Monoclonal
Inhibin A,P05111,cAb2812,Mouse,Monoclonal,cAb3150,Mouse,Monoclonal
Inhibin B,P05111,cAb2812,Mouse,Monoclonal,cAb2779,Mouse,Monoclonal
IRAK4,Q9NWZ3,cAb500,Rabbit,Monoclonal,cAb501,Rabbit,Monoclonal
KDR,P35968,cAb980,Rabbit,Monoclonal,cAb981,Rabbit,Monoclonal
KITLG,P21583,cAb2316,Rabbit,Monoclonal,cAb2314,Rabbit,Monoclonal
LAG3,P18627,cAb989,Rabbit,Monoclonal,cAb988,Rabbit,Monoclonal
LAMP3,Q9UQV4,cAb1931,Rabbit,Monoclonal,cAb1930,Rabbit,Monoclonal
LCN2,P80188,cAb3393,Mouse,Monoclonal,cAb3626,Mouse,Monoclonal
LGALS9,O00182,cAb757,Rabbit,Monoclonal,cAb2309,Rabbit,Monoclonal
LIF,P15018,cAb4295,Mouse ,Monoclonal,cAb3818,Mouse,Monoclonal
LTBR,P36941,cAb4106,Goat,Polyclonal,cAb2911,Mouse,Monoclonal
MDK,P21741,cAb1709,Rabbit,Monoclonal,cAb1708,Rabbit,Monoclonal
MERTK,Q12866,cAb816,Rabbit,Monoclonal,cAb815,Rabbit,Monoclonal
MET,P08581,cAb3822,Rabbit,Monoclonal,cAb3283,Mouse,Monoclonal
MICA,Q29983,cAb3164,Mouse,Monoclonal,cAb4215,Mouse,Monoclonal
MICB,Q29980,cAb3407,Rabbit,Monoclonal,cAb3638,Rabbit,Monoclonal
MIF,P14174,cAb570,Rabbit,Monoclonal,cAb1524,Rabbit,Monoclonal
MMP10,P09238,cAb2505,Mouse,Monoclonal,cAb2509,Mouse,Monoclonal
MMP12,P39900,cAb760,Rabbit,Monoclonal,cAb761,Rabbit,Monoclonal
MMP3,P08254,cAb3412,Rabbit,Monoclonal,cAb3643,Rabbit,Monoclonal
MMP8,P22894,cAb548,Rabbit,Monoclonal,cAb549,Rabbit,Monoclonal
MMP9,P14780,cAb1711,Rabbit,Monoclonal,cAb1713,Rabbit,Monoclonal
MPO,P05164,cAb3826,Mouse ,Monoclonal,cAb3827,Mouse,Monoclonal
MUC16,Q8WXI7,cAb670,Rabbit,Monoclonal,cAb671,Rabbit,Monoclonal
NCR1,O76036,cAb673,Rabbit,Monoclonal,cAb672,Rabbit,Monoclonal
NGF,P01138,cAb2161,Rabbit,Monoclonal,cAb1950,Rabbit,Monoclonal
OSM,P13725,cAb616,Rabbit,Monoclonal,cAb617,Rabbit,Monoclonal
PDCD1,Q15116,cAb3410,Rabbit,Monoclonal,cAb3641,Rabbit,Monoclonal
PDGFA,P04085,cAb938,Rabbit,Monoclonal,cAb3534,Mouse,Monoclonal
PTX3,P26022,cAb1734,Rabbit,Monoclonal,cAb1735,Rabbit,Monoclonal
S100A12,P80511,cAb1743,Rabbit,Monoclonal,cAb1742,Rabbit,Monoclonal
S100A9,P06702,cAb1746,Rabbit,Monoclonal,cAb1747,Rabbit,Monoclonal
SCG2,P13521,cAb1296,Rabbit,Monoclonal,cAb1295,Rabbit,Monoclonal
SELE,P16581,cAb1748,Rabbit,Monoclonal,cAb1749,Rabbit,Monoclonal
SLURP1,P55000,cAb4438,Rabbit,Polyclonal,cAb2419,Mouse,Monoclonal
SPP1,P10451,cAb1763,Rabbit,Monoclonal,cAb1762,Rabbit,Monoclonal
TAFA5,Q7Z5A7,cAb4077,Rat,Monoclonal,cAb2833,Goat,Monoclonal
TGFB1,P01137,cAb2028,Mouse,Monoclonal,cAb3937,Mouse,Monoclonal
TGFB3,P10600,cAb3884,Rabbit,Monoclonal,cAb3885,Rabbit,Monoclonal
THPO,P40225,cAb3657,Goat,Polyclonal,cAb1771,Rabbit,Monoclonal
TIMP1,P01033,cAb345,Rabbit,Monoclonal,cAb344,Rabbit,Monoclonal
TIMP2,P16035,cAb3413,Rabbit,Monoclonal,cAb3644,Rabbit,Monoclonal
TLR3,O15455,cAb1490,Rabbit,Monoclonal,cAb1491,Rabbit,Monoclonal
TNF,P01375,cAb4576,Mouse ,Monoclonal,cAb4065,Mouse,Monoclonal
TNFRSF11A,Q9Y6Q6,cAb1198,Rabbit,Monoclonal,cAb1197,Rabbit,Monoclonal
TNFRSF13C,Q96RJ3,cAb2916,Rabbit,Monoclonal,cAb2062,Goat,Polyclonal
TNFRSF14,Q92956,cAb3889,Rabbit,Monoclonal,cAb3890,Rabbit,Monoclonal
TNFRSF17,Q02223,cAb3891,Rabbit,Monoclonal,cAb3892,Rabbit,Monoclonal
TNFRSF18,Q9Y5U5,cAb3303,Rabbit,Monoclonal,cAb3304,Rabbit,Monoclonal
TNFRSF1A,P19438,cAb1776,Rabbit,Monoclonal,cAb1777,Rabbit,Monoclonal
TNFRSF1B,P20333,cAb3414,Rabbit,Monoclonal,cAb3645,Rabbit,Monoclonal
TNFRSF4,P43489,cAb3415,Rabbit,Monoclonal,cAb3646,Rabbit,Monoclonal
TNFRSF8,P28908,cAb1778,Rabbit,Monoclonal,cAb1779,Rabbit,Monoclonal
TNFRSF9,Q07011,cAb3928,Mouse,Monoclonal,cAb3110,Rabbit,Monoclonal
TNFSF10,P50591,cAb3918,Mouse,Monoclonal,cAb4188,Mouse,Monoclonal
TNFSF11,O14788,cAb3186,Mouse,Monoclonal,cAb3188,Rabbit,Monoclonal
TNFSF12,O43508,cAb1783,Rabbit,Monoclonal,cAb1782,Rabbit,Monoclonal
TNFSF13B,Q9Y275,cAb4371,Goat,Polyclonal,cAb2864,Rabbit,Monoclonal
TNFSF15,O95150,cAb1803,Rabbit,Monoclonal,cAb1802,Rabbit,Monoclonal
TNFSF4,P23510,cAb625,Rabbit,Monoclonal,cAb624,Rabbit,Monoclonal
TNFSF8,P32971,cAb3416,Rabbit,Monoclonal,cAb3647,Rabbit,Monoclonal
TNFSF9,P41273,cAb3417,Rabbit,Monoclonal,cAb3648,Rabbit,Monoclonal
TREM1,Q9NP99,cAb2103,Mouse,Monoclonal,cAb3296,Rabbit,Monoclonal
TSLP,Q969D9,cAb323,Mouse,Monoclonal,cAb3624,Mouse,Monoclonal
VCAM1,P19320,cAb1786,Rabbit,Monoclonal,cAb1787,Rabbit,Monoclonal
VEGFA,P15692,cAb3253,Rabbit,Monoclonal,cAb3252,Mouse,Monoclonal
VEGFC,P49767,cAb3195,Rabbit,Polyclonal,cAb3193,Goat,Monoclonal
VEGFD,O43915,cAb1788,Rabbit,Monoclonal,cAb1789,Rabbit,Monoclonal
VSNL1,P62760,cAb520,Rabbit,Monoclonal,cAb521,Rabbit,Monoclonal

VSTM1,Q6UX27,cAb2337,Rabbit,Monoclonal,cAb2338,Mouse,Monoclonal
WNT16,Q9UBV4,cAb1791,Rabbit,Monoclonal,cAb1790,Rabbit,Monoclonal
WNT7A,O00755,cAb774,Rabbit,Monoclonal,cAb775,Rabbit,Monoclonal

Validation

Antibodies were validated for NULISA applications. The best performing antibody pair was selected based on signal, background, specificity and endogenous protein detectability.
